# Early transcriptional response of human ovarian and fallopian tube surface epithelial cells to norepinephrine

**DOI:** 10.1038/s41598-018-26670-4

**Published:** 2018-05-29

**Authors:** Anxhela Gjyshi, Sweta Dash, Ling Cen, Chia-Ho Cheng, Chaomei Zhang, Sean J. Yoder, Jamie K. Teer, Guillermo N. Armaiz-Pena, Alvaro N. A. Monteiro

**Affiliations:** 1 Cancer Epidemiology Program, H. Lee Moffitt Cancer Center and Research Institute, Tampa, FL USA; 2 Cancer Biology PhD Program, University of South Florida, Tampa, FL USA; 3 Cancer Informatics Shared Facility, H. Lee Moffitt Cancer Center and Research Institute, Tampa, FL USA; 4 Molecular Genomics Shared Facility H. Lee Moffitt Cancer Center and Research Institute, Tampa, FL USA; 5 Cancer Biology Division, Ponce Research Institute and Department of Basic Sciences, Pharmacology Division, Ponce Health Sciences University, Ponce, PR USA

## Abstract

Evidence from human and animal studies suggests that chronic behavioral stress and resulting activation of the sympathetic nervous system may influence initiation and progression of tumors. However, the underlying mechanisms for these observations are poorly understood. The purpose of this study is to explore the effects of adrenergic signaling on cell line models derived from normal cells presumed to originate epithelial ovarian cancers. Here we explored the effects of the stress-related hormone, norepinephrine, on the transcriptional program of normal immortalized ovarian (iOSE) and fallopian tube (iFTSEC) surface epithelial cells. Analysis of RNA-Seq data of treated and untreated cells revealed a significant overlap between the responses in iOSE and iFTSEC cells. Most genes modulated by norepinephrine in ovarian and fallopian tube epithelial cells are already expressed in normal ovarian and fallopian tissue and cells. For several genes, expression changes were reflected at the protein level. Genes in immune-related and developmental pathways were enriched in the set of genes modulated by norepinephrine. We identified HOXA5, SPIB, REL, SRF, SP1, NFKB1, MEF2A, E2F1, and EGR1 transcription factor binding sites to be highly enriched in our dataset. These data represent the early transcriptional response to norepinephrine in cells postulated to originate epithelial ovarian cancer.

## Introduction

Clinical and epidemiological studies suggest that cancer onset and progression are associated with chronic stress, depression and other social and psychological factors^1–3^. It is well known that behavioral stress can activate the sympathetic nervous system, resulting in the release of catecholamines such as norepinephrine (NE) and epinephrine (Epi). Prolonged exposure to these stress-related hormones may negatively impact many physiological systems, including dysregulation of the cardiovascular system, cellular immune function and cancer risk^4–10^. At the molecular level, NE and Epi can induce β-2 adrenergic receptor (ADRB) activation and cyclic AMP levels and activate key pro-tumoral processes.

Growing evidence suggests that some adrenergic effects on cancer are independent of their influence on the immune system^11–13^. Catecholamines have been suggested to influence progression of solid tumors by inducing expression of pro-angiogenic and pro-metastatic factors, such as vascular endothelial growth factor (VEGF) and matrix metalloproteinases (MMPs)^11,13,14^. Chronic adrenergic stimulation, mediated primarily by NE, has been shown to induce inflammation, increase angiogenesis, prevent anoikis, promote tumor metastasis and also impair the efficacy of chemotherapeutic agents^13,15–19^. NE has been shown to enhance metastasis of breast, ovarian, prostate, colon and nasopharyngeal carcinoma tumor cells in *in vitro* studies and in pre-clinical *in vivo* models^13,14,20–22^. Interestingly, in human subjects chronic stress has been linked to elevated levels of systemic NE^23–28^ and a modest increase in ovarian cancer risk^29,30^.

Although, all major catecholamines are present in the ovary^31,32^, NE is the most abundant and plays an important role in ovarian steroidogenesis and follicular development^33–35^. Previous studies have assessed the effect of NE on ovarian cancer cell transcriptomic changes and response to chemotherapeutic agents^15,17,36–38^. While these studies reveal a role for NE in cancer progression, its role in cancer initiation remains underexplored or focused on immunologic aspects of the stress response. Here, we study the early transcriptional response to NE in two well-characterized normal immortalized cell lines derived from ovarian surface epithelium and fallopian tube surface epithelium^39,40^. These cells constitute a model representative of the presumed cells of origin of most high-grade serous ovarian cancers and have been used in several studies of functional analysis of susceptibility loci for ovarian cancer risk^41–48^.

## Results

### Differentially expressed genes identified by RNA-Seq

**Figure 1:**
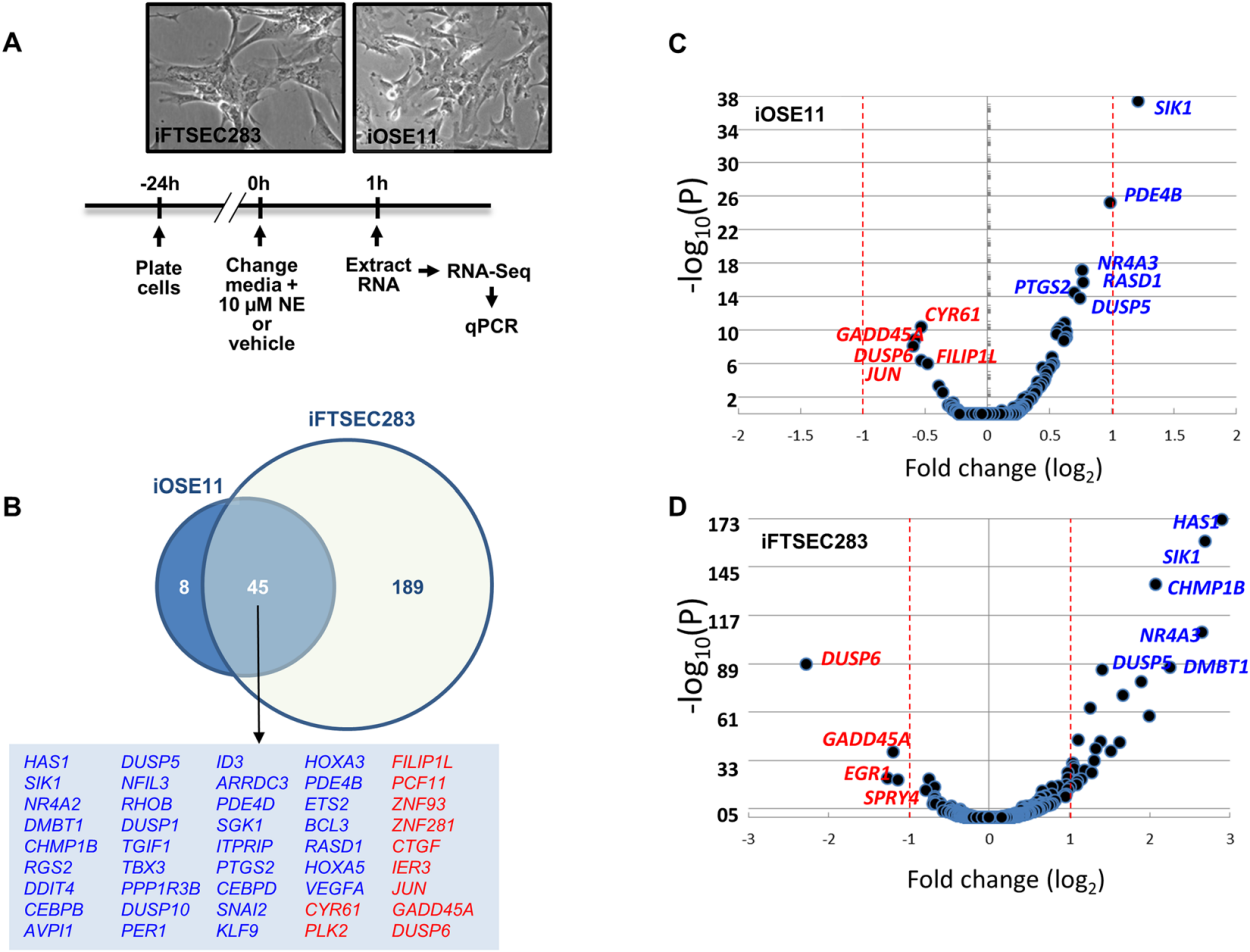
Early transcriptional response to norepinephrine. (**A**) Experimental timeline. (**B**) Venn diagram showing genes differentially expressed in response to norepinephrine (NE) treatment in iOSE11 and iFTSEC283 cells. Upregulated and downregulated genes are depicted in blue and red fonts, respectively. (**C**) and (**D**) Volcano plots for RNA-Seq data in iOSE11 (**C**) and iFTSEC283 (**D**).

To explore the effect of norepinephrine (NE) on cells postulated to be the precursors of epithelial ovarian cancer, we compared the transcriptome of immortalized ovarian surface epithelial and fallopian tube secretory epithelial cells (iOSE11 and iFTSEC283) mock-treated or treated with 10 µM NE for 1 h (Fig. 1A). This time point (1 h) was chosen based on a previous study on ovarian cancer cells in which significant changes in gene expression were found after 1 h of NE exposure^17^.

We identified a total of 53 and 234 differentially (False Discovery Rate < 0.1) expressed genes in iOSE11 and iFTSEC283 cells, respectively, upon NE treatment (Supplementary Tables 1 and 2). Forty-five of these genes were significantly (FDR < 0.1) differentially expressed and in the same direction (34 up and 11 down-regulated) in both cells lines (Fig. 1B–D) (Tables 1 and 2) (Supplementary Table 3). Next, we focused our attention on genes that have at least a 2-fold change in expression (FDR < 0.1 & |log_2_FoldChange| > 1) (Fig. 1C–D). While in the iOSE11 cell line, only one gene, (Salt-inducible kinase 1, *SIK1*) out of 45 was identified to have at least a 2-fold change in expression (p_adj_ = 3.75 × 10^−38^), we identified 29 of the 234 differentially expressed genes in the iFTSEC283 cell line (Table 1). Although not modulated to the same extent, 19 of these 29 genes were also significantly expressed in iOSE11 (Table 1).

**Table 1:** Upregulated genes.

Gene	Fold Change (log2) iFTSEC283	padj. iFTSEC283	Fold Change (log2) iOSE11	padj. iOSE11	Notes
*HAS1*	2.90	5.29E-173	0.29	0.0176649	
***SIK1***	2.69	1.72E-160	1.21	3.75E-38	Modulated after acute endurance exercise (in skeletal muscle)^53^
***NR4A2***	2.65	7.00E-108	0.58	5.24E-11	Ovary is the GTEx tissue in which it is most highly expressed. Most highly induced gene after acute endurance exercise (in skeletal muscle)^53^. Induced by adrenergic signaling in many tissues^56^.
***DMBT1***	2.25	1.30E-87	0.62	1.01E-09	
***CHMP1B***	2.07	1.42E-135	0.62	1.30E-11	
***RGS2***	2.00	1.25E-59	0.56	2.95E-10	Modulated after acute endurance exercise (in skeletal muscle)^53^
*DDIT4*	1.90	3.15E-79	0.26	0.088004	Ovary is the GTEx tissue in which it is most highly expressed.
***CEBPB***	1.67	2.32E-71	0.64	1.66E-10	Upregulated in response to NE in rat pineal gland^50^.
*AVPI1*	1.62	1.92E-44	0.50	2.72E-06	
*DUSP5*	1.41	3.49E-86	0.70	3.32E-15	Differentially expressed (≥2-fold) in ovarian carcinomas from patients with high biobehavioral risk (high depressive symptoms and low social support) vs. minimal biobehavioral risk^55^.
***NFIL3***	1.39	6.19E-45	0.74	1.61E-14	
*RHOB*	1.32	1.04E-40	0.42	0.000597549	
***DUSP1***	1.26	3.28E-64	0.56	1.39E-10	Upregulated in response to NE in rat pineal gland and in ovarian cancer cell lines^17,50^. Differentially expressed (≥2-fold) in ovarian carcinomas from patients with high biobehavioral risk (high depressive symptoms and low social support) vs. minimal biobehavioral risk^55^. Induced by stress in mouse hippocampus^54^.
*TGIF1*	1.15	3.64E-23	0.44	0.00032262	
*TBX3*	1.10	1.95E-45	0.50	3.57E-06	
*PPP1R3B*	1.10	1.32E-21	0.39	0.003632655	
*DUSP10*	1.05	1.95E-30	0.53	9.30E-07	
*PER1*	0.98	1.07E-23	0.33	0.027120352	Ovary is the GTEx tissue in which it is most highly expressed. Induced by stress in mouse hippocampus^54^. Induced by NE in glioma cells^51^.
*ID3*	0.98	1.58E-26	0.52	1.72E-07	
*ARRDC3*	0.96	2.84E-14	0.63	6.63E-10	Ovary is the GTEx tissue in which it is most highly expressed.
*PDE4D*	0.95	5.22E-13	0.52	1.18E-06	
*SGK1*	0.86	7.89E-19	0.39	0.001041981	Ovary is the GTEx tissue in which it is most highly expressed. Induced by stress in mouse hippocampus^54^. Induced by NE in HeyA8 ovarian cancer cells (GSE34405)
*ITPRIP*	0.78	2.92E-18	0.47	9.27E-06	
*PTGS2*	0.74	1.09E-07	0.76	2.02E-16	
*CEBPD*	0.68	9.51E-07	0.47	1.98E-05	Ovary is the GTEx tissue in which it is most highly expressed.
*SNAI2*	0.66	4.53E-16	0.40	0.000146807	
*KLF9*	0.64	1.34E-12	0.37	0.002381545	
*HOXA3*	0.61	1.65E-05	0.39	0.00239018	Fallopian tube is the GTEx tissue in which it is most highly expressed.
*PDE4B*	0.55	0.00043464	0.98	5.31E-26	
*ETS2*	0.54	1.46E-06	0.36	0.01274245	
*BCL3*	0.49	0.000686577	0.31	0.086790166	
*RASD1*	0.43	3.66E-05	0.76	7.47E-18	
*HOXA5*	0.42	0.010079643	0.45	0.000125642	Fallopian tube is the GTEx tissue in which it is most highly expressed.
*VEGFA*	0.41	6.81E-07	0.32	0.010595763	Increased expression in tumors of stressed animals^16^. Reduced by NE in neuroblastoma cells^52^.

**Table 2:** Downregulated genes.

Gene	Fold Change (log2) iFTSEC283	padj. iFTSEC283	Fold Change (log2) iOSE11	padj. iOSE11	Notes^b^
*CYR61*	−0.36	6.37E-05	−0.53	4.07E-11	Fallopian tube is the GTEx tissue in which it is most highly expressed. Induced by stress in mouse hippocampus^54^. Differentially expressed (≥2-fold) in ovarian carcinomas from patients with high biobehavioral risk (high depressive symptoms and low social support) vs. minimal biobehavioral risk^55^. Modulated after acute enduring exercise (in skeletal muscle)^53^
*PLK2*	−0.43	0.000124893	−0.36	0.002921785	
*FILIP1L*	−0.49	1.57E-06	−0.53	4.07E-07	
*PCF11*	−0.51	0.000295374	−0.31	0.085383988	Fallopian tube is the GTEx tissue in which it is most highly expressed.
*ZNF93*	−0.52	0.000347199	−0.29	0.094553363	
***ZNF281***	−0.61	2.21E-09	−0.39	0.000492736	
*CTGF*	−0.68	4.21E-13	−0.29	0.086790166	Differentially expressed (≥2-fold) in ovarian carcinomas from patients with high biobehavioral risk (high depressive symptoms and low social support) vs. minimal biobehavioral risk^55^.
*IER3*	−0.68	7.54E-12	−0.28	0.05080772	Modulated after acute endurance exercise (in skeletal muscle)^53^ Differentially expressed (≥2-fold) in ovarian carcinomas from patients with high biobehavioral risk (high depressive symptoms and low social support) vs. minimal biobehavioral risk^55^.
*JUN*	−0.69	3.63E-10	−0.48	9.30E-07	Fallopian tube is the GTEx tissue in which it is most highly expressed. Differentially expressed (≥2-fold) in ovarian carcinomas from patients with high biobehavioral risk (high depressive symptoms and low social support) vs. minimal biobehavioral risk^55^. Induced by NE is neuroblastoma cells^52^.
***GADD45A***	−1.19	5.96E-39	−0.58	1.55E-09	
***DUSP6***	−2.28	2.38E-89	−0.60	9.03E-09	Differentially expressed (≥2-fold) in ovarian carcinomas from patients with high biobehavioral risk (high depressive symptoms and low social support) vs. minimal biobehavioral risk^55^.

^a^Gene, gene symbol; Genes in bold were tested by qPCR; Genes underlined were examined for protein changes; ^b^Indicates whether the gene a) was identified as been modulated by stress, adrenergic signaling, or norepinephrine treatment in available datasets or b) Fallopian tube or ovary was the GTEx tissue (out of 53) in which the gene is most highly expressed (GTEx; additional data in Supplementary Table 6).

We annotated all 45 genes according to their expression levels in ovary and fallopian tube tissues, and in the tissues in which they were most highly expressed using RNA-Seq data from the Genotype-Tissue Expression (GTEx) (www.gtexportal.org) project which provides a comprehensive atlas of gene expression from multiple normal human tissues (Supplementary Table 3)^49^. Six and five genes had the highest expression in ovary and fallopian tube tissue of all tissues studied in GTEx (n = 53), respectively. Only two genes (*DMBT1* and *PTGS2*) did not show detectable expression in ovary or fallopian tube tissue. Three genes (*IER3*, *JUN* and *HOXA5*) were not detectable in ovary but in the case of *HOXA5*, fallopian tube was the tissue in which it was most highly expressed (Supplementary Table 3).

As GTEx expression reflects an average of expression of the different cell types in the tissue, we further explored whether the top ten modulated (5 up and 5 down regulated) genes were also expressed in ovarian and fallopian tube epithelial cells. We used previously generated FAIRE-Seq (Formaldehyde Assisted Isolation of Regulatory Elements followed by sequencing) and ChIP-Seq (Chromatin immunoprecipitation followed by sequencing) for Histone H3 Lysine 27 Acetylation (H3K27Ac; associated with active transcription) and Histone H3 Lysine 4 Monomethylation (H3K4me1; associated with the promoters of active genes) in iOSE4, iOSE11, iFTSEC33, and iFTSEC246^42^ to infer transcriptional activity (Supplementary Fig. 1). Eight of the ten genes examined showed strong evidence for transcriptional activity judging by the combination of H3K27Ac and H3K4me1 in ovarian and fallopian tube surface epithelia cells, while one (*HAS1*) displayed evidence for activity in ovarian but not fallopian tube surface epithelial cells (Supplementary Fig. 1). One gene (*DMBT1*) showed weak evidence for activity, with no detectable H3K27Ac. No significant eQTL association was found for any gene in ovarian or fallopian tube tissues. Taken together, these results suggest that most genes modulated by norepinephrine in ovarian and fallopian tube surface epithelial cells are already actively expressed in normal ovarian and fallopian tissue and cells (Table 1).

Fourteen unique genes had been previously shown to be modulated by NE (n = 6)^50–52^, stress (n = 12)^16,53–55^, or adrenergic signaling (n = 1)^56–58^ in other tissues such as the human skeletal muscle, mouse hippocampus, rat pineal glands, or neuroblastoma and ovarian cancer cells^17,50,52–54^ (Table 1), suggesting that a significant fraction of target genes (14/45; 31%) are common targets in multiple tissues.

To investigate the association between these 45 genes and ovarian cancer we analyzed data from the Cancer Genome Atlas (TCGA) for ovarian serous cystadenocarcinoma (TCGA, Provisional) from tumor samples with mRNA data (Agilent microarray) (n = 558 samples). While no gene had significant rates of somatic point mutations (all ≤ 0.5%) (Supplementary Table 3), 88% of all samples displayed alterations in the 45 genes, with 36 genes showing a high frequency (>5%) of alterations (amplifications, deletions, mRNA up or down regulation) (Supplementary Fig. 2). Interestingly, the predominant alterations were amplification and mRNA upregulation, with only *CHMP1B*, *PPP1R3B*, *ARRDC3*, *PDE4D*, *RASD1*, and *PLK2* showing a preponderance of deep deletions and mRNA downregulation. No particular association with fractional genome alterations, age of diagnosis, or mutation spectrum was seen. These data suggest that there is no strong association between the genes responsive to NE in ovarian and fallopian tube surface epithelial cells and somatic alterations in ovarian epithelial cancer.

### Verification of differentially expressed genes by qPCR and western blotting

**Figure 2:**
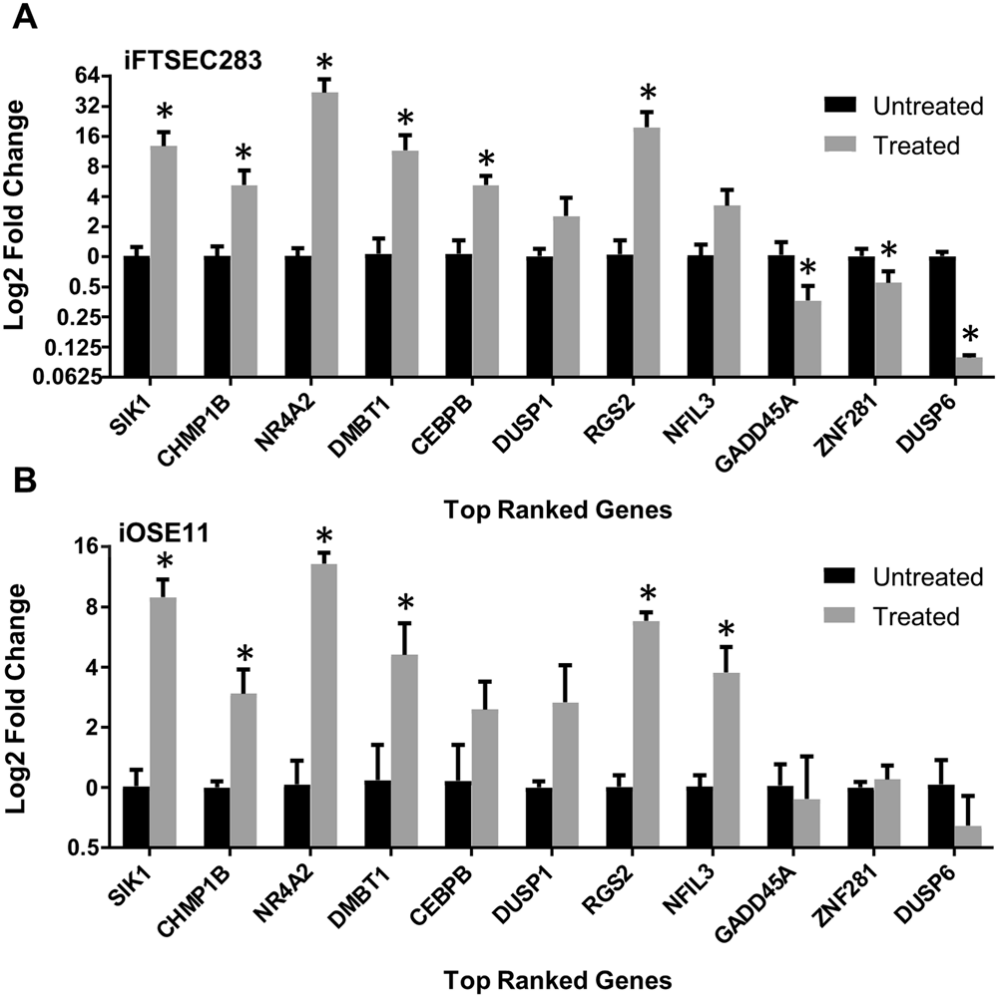
qPCR confirmation of expression changes. Gene expression as measured by RT-qPCR in iOSE11 (**A**) or iFTSEC283 (**B**) treated with norepinephrine for eight and two upregulated and downregulated genes, respectively. Expression is plotted as log2 fold change in comparison to the mock-treated cells.

The top ranked differentially expressed genes that were identified by RNA-Seq were further analyzed for verification by RT-qPCR. We tested eight upregulated genes (*SIK1*, *CHMP1B*, *NR4A2*, *DMBT1*, *CEBPB*, *DUSP1*, *RGS2*, and *NFIL3*) and three that were downregulated in both cell lines (*GADD45A*, *DUSP6*, and *ZNF281*) (Table 1). We confirmed that all genes shown to be modulated by NE treatment using RNA-seq were also modulated in the same direction when verified by RT-qPCR in both cell lines (Fig. 2). While most genes displayed a statistically significant difference (p ≤ 0.05), a few did not, although the direction of change was consistent with the RNA-Seq results.

**Figure 3:**
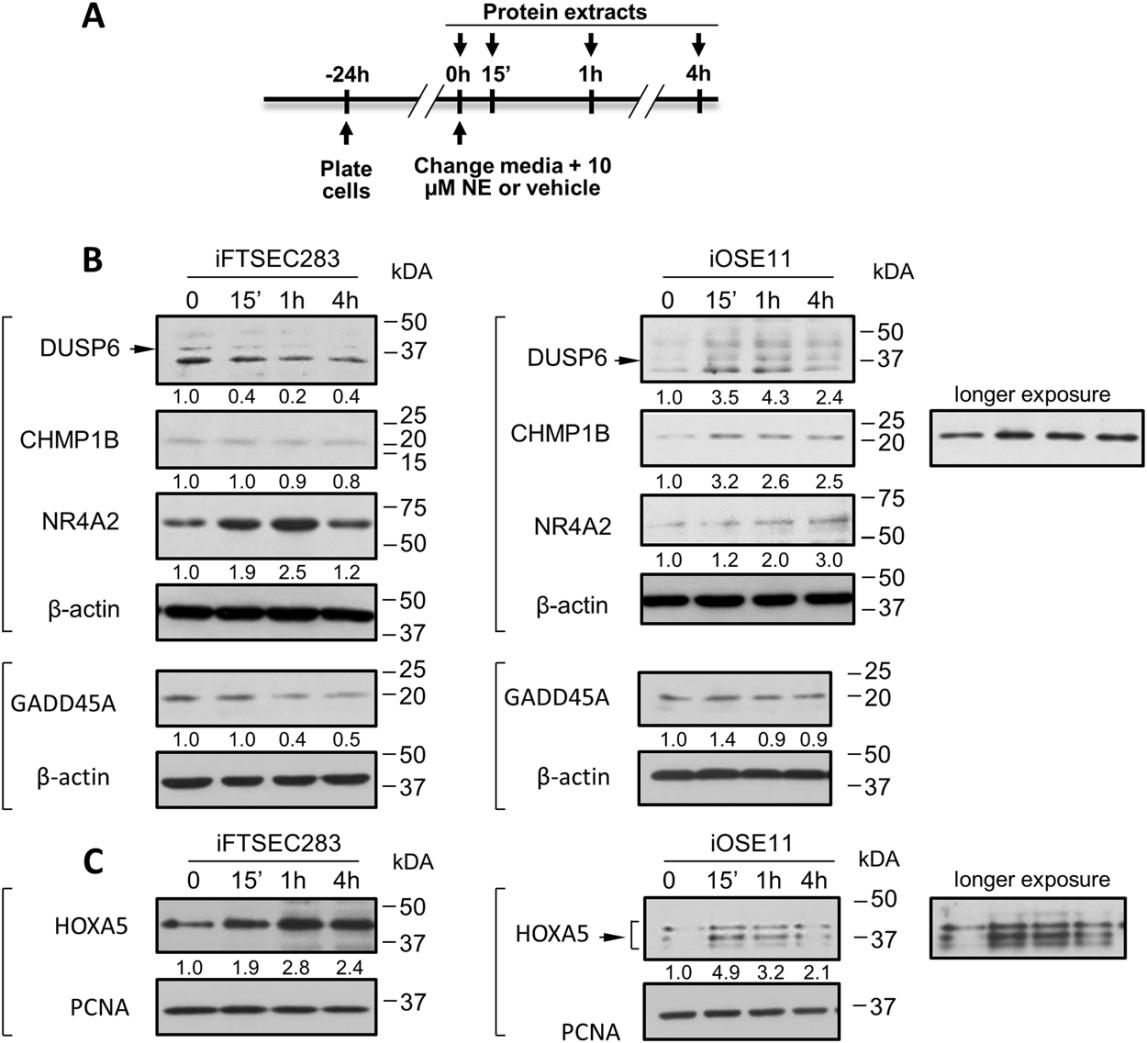
Analysis of protein level changes upon norepinephrine treatment. (**A**) Experimental timeline. (**B**) Western blot analysis of FTSEC283 and iOSE11 cells. Lysates were separated by PAGE, transferred to PVDF and blotted with antibodies against the indicated proteins. Β-actin was used as a loading control. (**C**) Western blot analysis of FTSEC283 and iOSE11 cells. Nuclear extracts were separated by PAGE, transferred to PVDF and blotted with antibodies against HOXA5. PCNA was used as a loading control. The arrows indicate the expected band corresponding to the protein in question. Numbers below blots are densitometric measurements and represent changes in relation to the 0 h control. Measurements are normalized to their respective internal controls (β-actin or PCNA).

To investigate the extent to which changes in gene expression were reflected in changes at the protein level we determined steady-state levels of DUSP6, GADD45A, CHIMP1B and NR4A2 in iOSE11 and iFTSEC283 cells following NE treatment for 15 min, 1 h and 4 h by western blotting using β-actin as an internal loading control (Fig. 3A) to generate normalized densitometry measurements. DUSP6 (~42 kDa) and GADD45A (~18 kDa) protein levels decreased with increasing time of NE exposure in iFTSEC283 but were virtually unchanged or, in the case of DUSP6 increased (Fig. 3B) in iOSE11 reflecting changes observed in qPCR (Fig. 2). CHMP1B up regulation at the protein level was only observed in iOSE11. NR4A2 increase in protein level upon NE treatment was observed in both cell lines reflecting changes in transcript levels (Fig. 3). Interestingly, while levels progressively increase with time of treatment in iOSE11 cells, a peak at 1 h was observed in iFTSEC283 with a return to lower levels at 4 h. In summary several genes identified showed corresponding changes in protein levels as those observed by RNA-Seq and confirmed by qPCR.

### Pathway Analysis

Three Panther pathways were significantly (p_adj_ < 0.05) overrepresented: Oxidative stress response (fold enrichment = 42.43; p_adj_ = 2.21 × 10^−5^), p38 MAPK pathway (fold enrichment = 34.15; p_adj_ = 1.59 × 10^−2^), and Gonadotropin-releasing hormone receptor pathway (fold enrichment = 9.89; p_adj_ = 2.41 × 10^−2^) (Supplementary Table 4).

The only Panther GO-Slim molecular function significantly (p_adj_ < 0.05) overrepresented was sequence-specific DNA binding transcription factor activity (fold enrichment = 4.4; p = 4.74 × 10^−03^). Consistent with this result, the only Panther Protein Class significantly overrepresented was the transcription factor class (fold enrichment = 4.05; p_adj_ = 1.16 × 10^−2^). When the analysis was restricted to the eleven downregulated genes, the only enrichment detected was of Panther biological process of cell death (fold enrichment = 29.30; p_adj_ = 4.28 × 10^−6^) (Supplementary Table 4).

To map the identified differentially expressed genes to potentially altered pathways, the MetaCore pathway analysis was performed to identify significant biological processes and pathways for the two cell types separately. In iFTSEC283 cells, The Developmental_Yap/TAZ-mediated (*i*.*e*. *EDN1*, *SLUG*, *SNAIL1*, *VEGFA*, *ID1*, *ID2*, *ID3*, and FKHR) co-regulation of transcription and Immune response IL-6 and IL-4 signaling pathways were the most significant interactions for the up-regulated genes (Supplementary Fig. 3A). For the iOSE11 cells, the Immune response IL-5 and IL-6 signaling pathways via JAK/STAT were the most significant (Supplementary Fig. 3B).

Common pathways that emerged from both cell lines include: the Immune response_IL-6 and IL-5 immune signaling pathways via JAK/STAT, Developmental_YAP/TAZ regulation of transcription pathway, and Signal transduction_PTMs in IL-17-induced CIKS-independent signaling pathway. For the down-regulated genes, the common pathways that emerged from both cell lines include the Regulation of Tissue Factor signaling in cancer, Immune response_IL-1 signaling pathway, Expression targets of Tissue factor signaling in cancer, and Immune response_Oncostatin M signaling via MAPK (Supplementary Fig. 1C,D). These data indicate that immune-related pathways are significantly modulated in ovarian cells in response to NE.

### Promoter Enrichment Analysis

To infer common gene regulatory mechanisms induced by norepinephrine, we explored enrichment of transcription factor binding sites (TFBS) in the 5 kb sequence up and downstream of transcription start sites of the 45 differentially expressed genes identified from the RNA-Seq data. In this analysis, we aim to identify transcription factors that might play a central and early role in the transcriptional response to NE.

This was done using oPOSSUM and a pre-computed JASPAR database of conserved TFBSs through computing two complementary statistical measures, Fisher exact test and Z-score^59^. Fisher scores which are based on a one-tailed Fisher exact probability assess the number of genes with the TFBS motifs in the foreground set vs. the background set. Z-scores are based on normal approximation to the binomial distribution that measures the change in the relative number of TFBS motifs in the foreground gene set compared with the background set.

**Figure 4:**
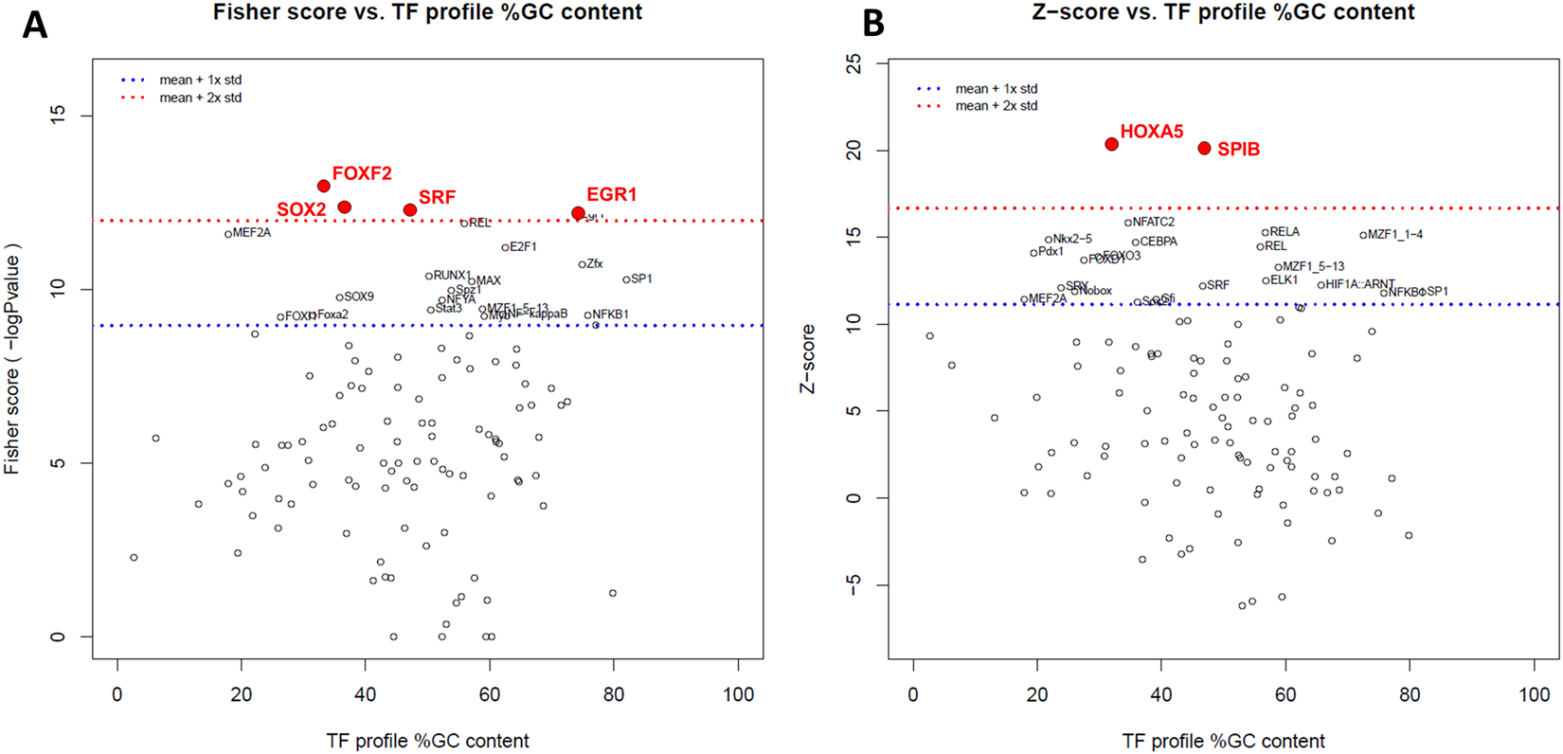
Transcription factors whose binding sites are enriched in the promoter of genes responsive to norepinephrine in ovarian and fallopian tube cells. Identification of enriched transcription factors using oPOSSUM. (**A**) Significantly enriched using Fisher score. (**B**) Significantly enriched using Z-score. Dashed blue and red lines represent one and two standard deviations above the mean, respectively. Scores (Z-score and Fisher score) are plotted vs. the GC composition of the TF profile. TF with scores higher than 2SD are highlighted in red font.

We identified 116 potential transcription factor binding sites that were enriched in our dataset and ranked them by Z- score and Fisher score (Supplementary Table 5, Fig. 4). When ranked by Z-scores, two transcription factors were found to have a Z-score higher than 2 standard deviations above the mean, HOXA5 and the ETS transcription factor SPIB. Ranking by Fisher’s score led to the identification of four transcription factors, FOXF2, SOX2, SRF, and EGR1.

Finally, we interrogated the extent to which levels of HOXA5 in the nucleus by western blotting using PCNA as an internal loading control (Fig. 3C). Levels consistently increased after NE treatment, peaking at 15′ and 1 h in iOSE11 and iFTSEC283, respectively. Note that iOSE11 cells present at least three isoforms recognized by the antibody against HOXA5. Thus, levels of HOXA5 also reflect the changes observed in the RNA-Seq and confirmed by qPCR.

## Discussion

To explore possible mechanisms by which stress could influence ovarian cancer initiation, we focused on early transcriptional changes induced in normal ovarian and fallopian tube surface epithelial cells by stress hormones. We compared the transcriptome of cells derived from ovarian (iOSE11) and fallopian tube surface epithelium (iFTSEC283), mock-treated and treated with 10 µM Norepinephrine for 1 h (Fig. 1).

We identified 45 genes that were significantly modulated in the same direction by norepinephrine (NE) in both cell lines. *SIK1* transcripts were significantly and robustly induced in both cell lines studied. The protein encoded by *SIK1* is a Class II HDAC Class II kinase^60^ and has been implicated in the regulation of hormonal signaling in adrenal and adipose tissues^61^. Interestingly, *SIK1* is altered in ~5% of ovarian high grade serous epithelial ovarian cancer in TCGA with most altered tumors displaying amplification or RNA upregulation.

This set of 45 genes is enriched with transcription factors, judging by gene ontology, and may represent the early wave of transcriptional response to norepinephrine. Notably, the set of genes down-regulated by NE is enriched in genes involved in cell death (*GADD45A*, *DUSP6*, *CTGF*, *CYR61*, *JUN*, and *IER*3). The significant overlap of genes differentially expressed in both cell lines is consistent with their common embryological origin and with a previous analysis of chromatin features^42^. Our data suggests that both cell types share a similar early transcriptional response to NE.

Analysis of cell and nuclear lysates for five select proteins (DUSP6, GADD45A, CHIMP1B, NR4A2, and HOXA5) demonstrated that, for the most part, changes observed by RNA-Seq and confirmed by qPCR were reflected in changes of steady-state protein levels. This indicates that these changes are likely to have implications for downstream cellular events in ovarian surface and fallopian tube epithelial cells.

Pathway analysis using Metacore revealed a significant enrichment in pathways involved in immune response, suggesting a possible cross-talk between ovarian/fallopian epithelial cells and the immune system. It may also reflect a common program of transcriptional responses that are not tissue specific. Eleven genes (11/45) were found to be significantly modulated by stress, adrenergic signaling, or NE in tissues other than ovary and fallopian tube, albeit not always in the same direction. For example, stress induces the expression of *Dusp1*, *Cyr61*, *Per1*, and *Sgk1* in mouse brain^54^, genes found to be significantly induced in iFTSEC283 and iOSE11 cells.

Several genes have already been implicated in ovarian cancer or in ovarian biology. For example, *DUSP1*, one of several dual specificity phosphatases in our set (*DUSP5*, *DUSP6*, and *DUSP10*), is involved in human cellular response to environmental stress and has been shown to induce chemoresistance in human ovarian cancer^17^. The homeotic transcription factor *HOXA5* was induced in ovarian and fallopian tube epithelial cells in response to NE. Whereas its expression is not detected in ovarian tissue, fallopian tube is the tissue in which it is most highly expressed (GTEx). Loss of *Hoxa5* leads to prolonged estrous cycle and ovarian epithelial inclusion cysts^62^. Interestingly, analysis of the promoter regions of genes significantly modulated by norepinephrine revealed that binding sites to HOXA5 were the most highly enriched, suggesting a central role for this factor in the response to NE. Importantly, these changes were also reflected in protein levels in the nucleus of iFTSEC283 and iOSE11.

Limitations of this study include the limited number of immortalized normal cell lines, the *in vitro* conditions that do not take into account complex cell-cell and cell-matrix interactions, and an acute exposure to a single dose of NE. Despite these limitations, our study is the first step in the systematic analysis of the effects of stress hormones on normal surface epithelial cells of the ovary and the fallopian tube. Future experiments using shorter and longer treatment times, co-culture of different cell types, and genetic manipulation of candidate transcription factors are likely to illuminate the mechanistic aspects of NE transcriptional response and its role in ovarian biology.

## Materials and Methods

### Cell lines

The cell lines used here were chosen as relevant models for ovarian cancer initiation. They represent the cell types postulated to serve as the originating cells for high grade serous epithelial ovarian cancer which can have contributions from different tissues (ovary surface epithelium and fallopian tube secretory epithelium) to epithelial ovarian cancer^63^.

We chose two established ovarian cell lines: immortalized normal ovarian surface epithelial cells, iOSE11^64^, and fallopian tube epithelial cells, iFTSEC283, and verified their expression of adrenergic receptors (ADRB1, ADRB2, and ADRB3). All three receptors were expressed at comparable levels in both cell lines (Supplementary Fig. 4). Cell lines were cultured in NOSE-CM Medium as described previously^39^. The medium is comprised of MCDB105 and Medium 199 (Sigma-Aldrich, St. Lois, MO) (1:1) supplemented with 15% fetal bovine serum (Sigma-Aldrich, St. Lois, MO), 10 ng/ml epidermal growth factor (Thermo Fisher Scientific, Waltham, MA), 0.5 mg/ml hydrocortisone (Sigma-Aldrich), 5 mg/ml insulin (Sigma-Aldrich), and 34 mg protein/ml bovine pituitary extract (Thermo Fisher Scientific, Waltham, MA). Cell line authentication was performed by the Molecular Genomics Core using the Promega GenePrint 10 short tandem repeat analysis system (Promega, Madison, WI) and mycoplasma testing was performed using the PCR Mycoplasma Detection Kit (Applied Biological Materials, Vancouver, Canada).

### RNA isolation

Cells were plated on 100 mm plates and cultured for 24 h after plating to reach 80% confluency. Cells were then briefly washed with PBS (Thermo Fisher Scientific) and fresh medium containing 10 µM norepinephrine (Sigma-Aldrich) or vehicle (H_2_O) and incubated for 1 h (Fig. 1A). Cells were harvested immediately after treatment and processed for total RNA extraction using the RNeasy Mini Kit (Qiagen, Hilde, Germany) following manufacturer’s protocol, including the optional “on-column DNase digestion” step using freshly prepared DNase. Disruption and homogenization of cells was performed using QIAshredder (Qiagen). Quality and purity of the RNA samples was tested by electrophoresis in a 1% agarose gel exhibiting sharp 28S and 18S rRNA bands and the ratio of absorbance at 260/230 was ≥2 as measured by Nanodrop. Reverse transcription was performed using the QuantiTect Reverse Transcription kit (Qiagen). RNA samples were used for sequencing and for verification of gene expression by qPCR.

### qPCR

Commercially available SYBR®Green-based PCR assays (Qiagen) were used for gene expression analysis of *ADRB1*, *ADRB2*, and *ADRB3* using *GAPDH* as an internal control. Expression for each gene of interest was calculated as a relative expression ratio normalized to *GAPDH* expression levels.

The same RNA samples that were sent to sequencing were also used for validation by RT-qPCR. cDNA samples were analyzed by real-time PCR on a 7900HT Fast Real-Time PCR System. Taqman assays (Thermo Fisher Scientific) were used to test *SIK1*, *CHMP1B*, *NR4A2*, *DMBT1*, *CEBPB*, *DUSP1*, *DUSP6*, *RGS2*, *NFIL3*, *GADD45A*, and *ZNF281* expression in treated and mock-treated samples for each cell line. 18S was used as an internal control. PCR reactions were set up in triplicates for each sample. The Δ-Δct method was used for the analysis. Data is presented as log_2_ fold change in reference to the untreated samples for each cell line.

### Library preparation and massively parallel sequencing

Total RNA was collected for each cell line and treatment condition in three independent replicates. 100 ng of total RNA was used for library preparation using Ovation Human FFPERNA-seq multiplex system (NuGEN Technologies, San Carlos, CA). Sequencing was performed on an Illumina NextSeq500 instrument with 75 bp paired end reads. On average, 23 million pairs of reads were generated for each sample. The average alignment rate was 92.5% with Q30 ≥ 94%.

### RNA-Seq Analysis

Following initial quality assessment and adaptor trimming, sequencing reads were aligned with Tophat v2.0.13^65^ against human reference genome hs37d5. Quantification of read counts aligned to the region associated with each gene was performed using HTSeq v0.6.1^66^ based on National Center for Biotechnology information (NCBI) RefSeq gene model. Read counts of all samples were normalized using the median-of-ratios method implemented in R/Bioconductor package DESeq2 v1.6.3^67^. Differential expression analysis between the two groups was performed by serial dispersion estimation and statistical model fitting procedures implemented in DESeq2. Genes with a p-value (adjusted for multiple testing with the Benjamini-Hochberg correction) of less than 0.1 (and/or a fold change of 2 and above) were determined to be significantly differentially expressed.

### Over-representation analysis of regulatory motif

oPOSSUM^59^ single site analysis was applied with the following options: 0.40 conservation cutoff, 85% matrix match threshold, sequences of −5,000 to 5,000 bp from the transcription start site, and all genes in the oPOSSUM database. Shared common genes with a p-value < 0.1 from the differential analysis in the two cell lines were used as an input to be searched against the Jaspar database.

### Gene ontology

PANTHER (version 12.0 Released 2017-07-10) Overrepresentation Test (release 20170413) was used to perform Gene Ontology (GO) enrichment analysis, applying the Bonferroni correction for multiple testing.

### Pathway analysis

The Pathway enrichment analysis was conducted using GeneGo MetaCore Software (Version 6.31 build 68930, Thomson Reuters). Significantly enriched biological processes and pathways were called by using a FDR value of less than 0.1. The p value and FDR were calculated for each pathway map in iFTSEC283 and iOSE11 cells for upregulated and downregulated differentially expressed genes.

### Annotation data

For tissue expression annotation we used the GTex Portal (www.gtexportal.org) with data from Release V7 (dbGaP Accession phs000424.v7.p2)^49^. Expression in ovary (n = 122) and fallopian tube (n = 7) samples was measured in transcripts per million (TPM). We also integrated FAIRE-Seq (Formaldehyde Assisted Isolation of Regulatory Elements followed by sequencing) and ChIP-Seq (Chromatin immunoprecipitation followed by sequencing) for Histone H3 Lysine 27 Acetylation and Histone H3 Lysine 4 Monomethylation in iOSE4, iOSE11, iFTSEC33, iFTSEC246, iFTSEC283^42^. To annotate somatic alterations in ovarian cancer we used Ovarian Serous Cystadenocarcinoma (TCGA, Provisional) tumor samples with mRNA data (Agilent microarray) (558 samples) via the cBioPortal (http://www.cbioportal.org) on November, 2017.

### Protein analysis

Cells were cultured to 80% confluency and treated with freshly prepared 10 µM NE or vehicle (H_2_O) at the following time points: 0 min, 15 min, 1 h and 4 h. After treatment cells were washed with PBS, harvested by scraping, and lysed with lysis buffer A (20 mM Tris pH 7.4, 10% glycerol, 10 mM KCL, 0.2% NP-40, 1 mM EDTA, 0.6 mM β-mercapto ethanol) supplemented with 1 mM PMSF and 1x protease inhibitor cocktail (Roche, Basel, Switzerland) for 2 min on ice. After centrifugation (12,000 rpm), the supernatant constituted the cytoplasmic fraction and pellets were resuspended in nuclear extract buffer B (20 mM Tris (pH 7.4), 20% glycerol, 10 mM KCL, 0.4 M NaCl, 1 mM EDTA, 0.6 mM β-mercapto ethanol, 1 mM PMSF and 1x protease inhibitor cocktail) for 30 min on ice. Protein concentration was determined using the Bradford Assay (Bio-Rad Laboratories, Hercules, California).

Nuclear cell extracts (40–50 µg protein) were used for immunoblotting HOXA5 (Sigma-Aldrich) and whole cell lysates containing cytoplasmic and nuclear fractions (40–50 µg protein) were resolved in 10% polyacrylamide gels and transferred to activated PVDF using the TransBlot Turbo system (Bio-Rad Laboratories, Hercules, California). Antibodies: α-HOXA5 (Sigma-Aldrich; HPA029319; dilution 1:1000), α-DUSP6 (Sigma-Aldrich; SAB1410312; dilution 1:1000), α-NR4A2 (Sigma-Aldrich; AV38753; dilution 1:1000), α-PCNA (Cell Signaling; 13110S; dilution 1:1000), α-β-Actin (Santa Cruz; sc-47778; dilution 1:1000), α-CHMP1B (Sigma-Aldrich; SAB2106113; dilution 1:3000), α-GADD45 (Sigma; SAB1405705; dilution 1:500). Secondary antibody conjugates: Goat α-Mouse IgG (H + L) HRP (Thermo Scientific; 31430) and Goat α-Rabbit IgG (H + L) HRP (Thermo Scientific; 31460). Membranes were blocked and probed with primary and secondary antibodies according to manufacturers’ suggested concentrations. Protein levels were interrogated in at least two independent experiments. Western blot densitometry was measured using NIH ImageJ (Imaging processing and analysis in Java) software. Values for each lane were first normalized to the 0 h NE treatment and then ratios were obtained for each treatment point by dividing the values of the protein of interest by the values of the internal control (β-actin or PCNA) (Supplementary Fig. 5).

### Data availability

The RNA-seq datasets (raw data, processed normalized count and meta-data) generated and analyzed during the current study are available through GEO Profiles (GSE108084).

## Electronic supplementary material

Supplementary Figures

Supplementary Tables 1-6
