# Supplementary material for: Early transcriptional response of human ovarian and fallopian tube surface epithelial cells to norepinephrine
Source: Sci Rep. 2018 May 29;8:8291. doi: 10.1038/s41598-018-26670-4 (PMC5974302; doi:10.1038/s41598-018-26670-4)
Supplement: Supplementary file 1 — Supplementary Figures [file 41598_2018_26670_MOESM1_ESM.pdf]

Supplementary Information for:

Early transcriptional response of human ovarian and fallopian tube surface epithelial cells to norepinephrine

Anxhela Gjyshi[1,2], Sweta Dash[1,2], Ling Cen[3], Chia-Ho Cheng[3], Chaomei Zhang[4], Sean J. Yoder[4], Jamie K. Teer[3], Guillermo N. Armaiz-Pena[5], Alvaro N.A. Monteiro[1*]

This file contains:

Supplementary Figure 1. Chromatin landscapes of 10 top modulated genes in response to norepinephrine. Each panel represents a screen shot of the UCSC Human Genome browser for each of the ten genes. Upregulated and downregulated genes are indicated by the blue and the red arrows, respectively. All panels are shown in the same scale. Twelve chromatin tracks are shown including four (iOSE4, iOSE11, iFTSEC33, iFTSEC246) each for FAIRE-seq (black bars), H3K27Ac (orange bars), and H3K4me1 (yellow bars). The red dashed box highlights the proximal promoter region.

Supplementary Figure 2. Oncoprint from the Cancer Geneome Atlas (TCGA) for the 45 genes responsive to norepinephrine. Each bar corresponds to a single case.

Supplementary Figure 3. Metacore pathway enrichment. Pathways enriched in iOSE11 **(A and C)** and iFTSEC283 **(B and D)** cells using the set of upregulated **(A and B)** or downregulated **(C and D)** genes**.**

Supplementary Figure 4. Expression of adrenergic receptors in iOSE11 and iFT283 cells. Expression of *ADRB1*, *ADRB2*, and *ADRB3* verified by qPCR in two technical replicates (orange or red circles) is plotted as a relative expression ratio normalized to *GAPDH* expression levels.

Supplementary Figure 5. Densitometry measurements. Densitometry measurements from western blots in Figure 3B and C using NIH Image J. A table with final calculations is shown with values that were used in Figure 3B and C (rounded to the first decimal position).

The following Supplementary Tables are provided as a separate Excel file:

Supplementary Table 1. Differentially (FDR < 0.1) regulated genes upon norepinephrine treatment in iOSE11. Gene, gene symbol; baseMeanIOS, mean of normalized counts for all the iOSE11 samples; log2FoldChange.IOS, $\log_2$ fold change in treated iOSE11 vs. control; lfcSE.IOS, standard error; stat.IOS, Wald statistic; pvalue.IOS: Wald test p-value; padj.IOS, Benjamini-Hochberg adjusted p-value. Genes highlighted in green and red are upregulated and downregulated, respectively.

Supplementary Table 2. Differentially (FDR < 0.1) regulated genes upon norepinephrine treatment in iFT283. Gene, gene symbol; baseMean.FT, mean of normalized counts for all the iFTSEC283 samples; log2FoldChange.FT, $\log_2$ fold change in treated iFTSEC283 vs. control; lfcSE.FT, standard error; stat.FT, Wald statistic; pvalue.FT: Wald test p-value; padj.FT, Benjamini-Hochberg adjusted p-value. Genes highlighted in green and red are upregulated and downregulated, respectively.

Supplementary Table 3. Annotation of forty-five differentially regulated genes upon norepinephrine treatment in both cell lines. GTEx Ovary (TPM), expression in transcripts per million in ovarian tissue from GTEx; GTEx Fallopian (TPM), expression in transcripts per million in ovarian tissue from GTEx; GTEx tissue highest expression (TPM), tissue demonstrating the highest expression in transcripts per million; GTEx Ovary rank, ranking of ovary within GTEx tissues according to expression in TPM, only annotated up to the fifth tissue in the rank; GTEx Fallopian rank, GTEx Ovary rank, ranking of ovary within GTEx tissues

according to expression in TPM, only annotated up to the fifth tissue in the rank. Somatic Point Mutation Freq (%), missense, nonsense and frameshift point mutations in TCGA for ovarian cystadenocarcinoma; References, articles implicating the gene in the response to Epinephrine, Adrenergic signaling or stress; GEO Datasets, publically available datasets.

Supplementary Table 4. Panther Gene Ontology analysis.

Supplementary Table 5. Enriched transcription factor binding site.

Supplementary Table 6. Dataset annotation.

Supplementary Figure 1

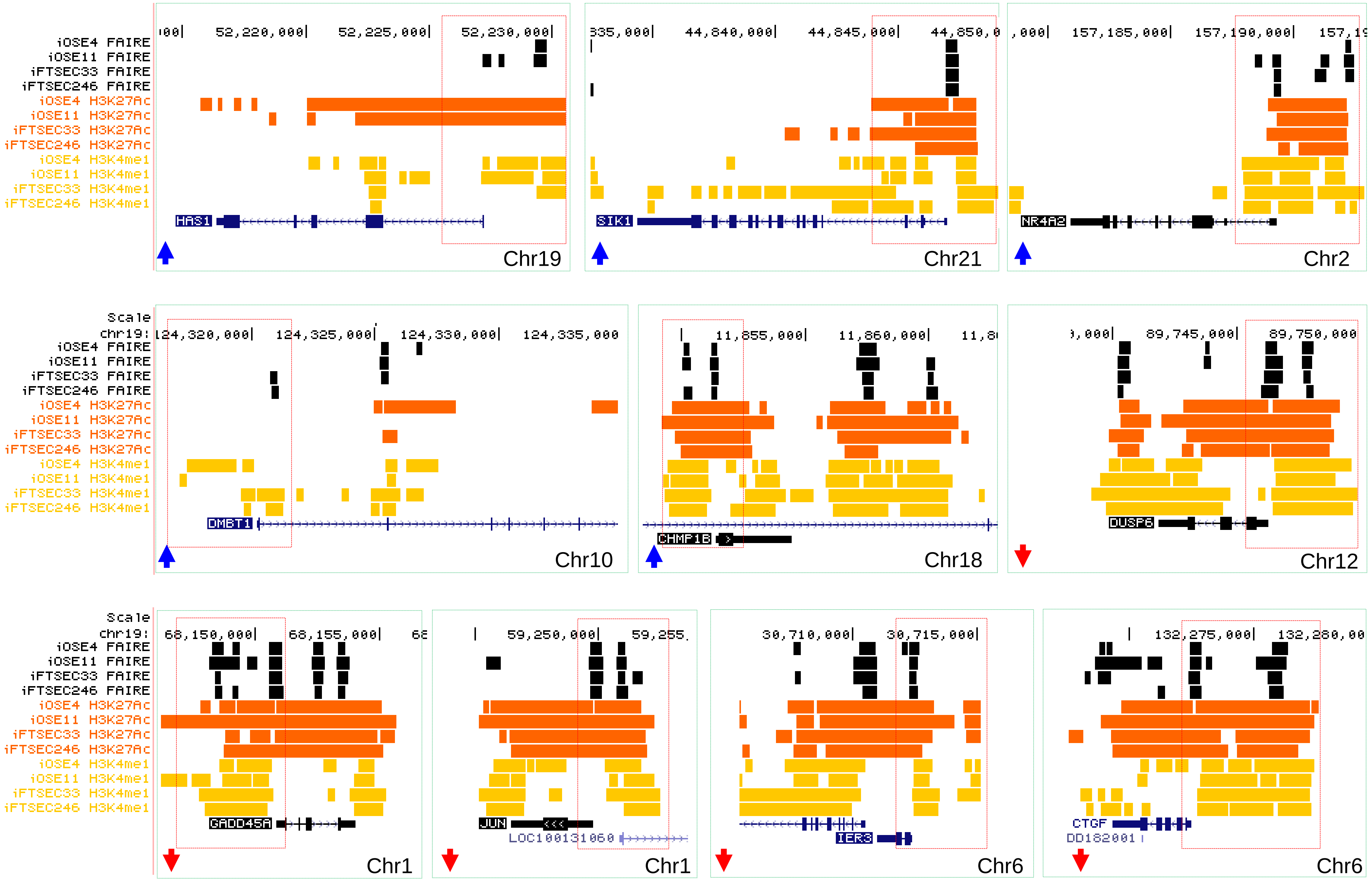

Supplementary Figure 2

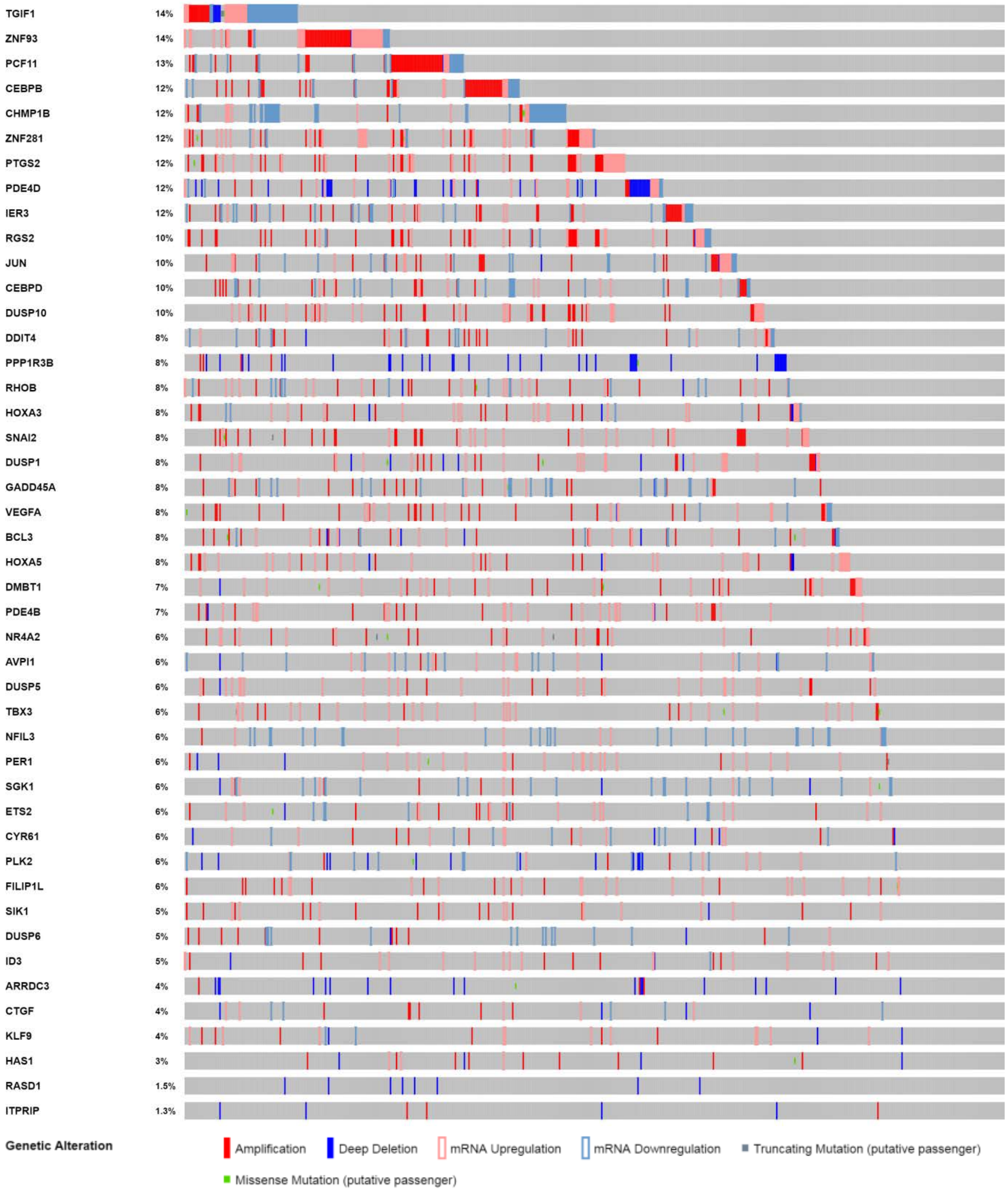

Supplementary Figure 3

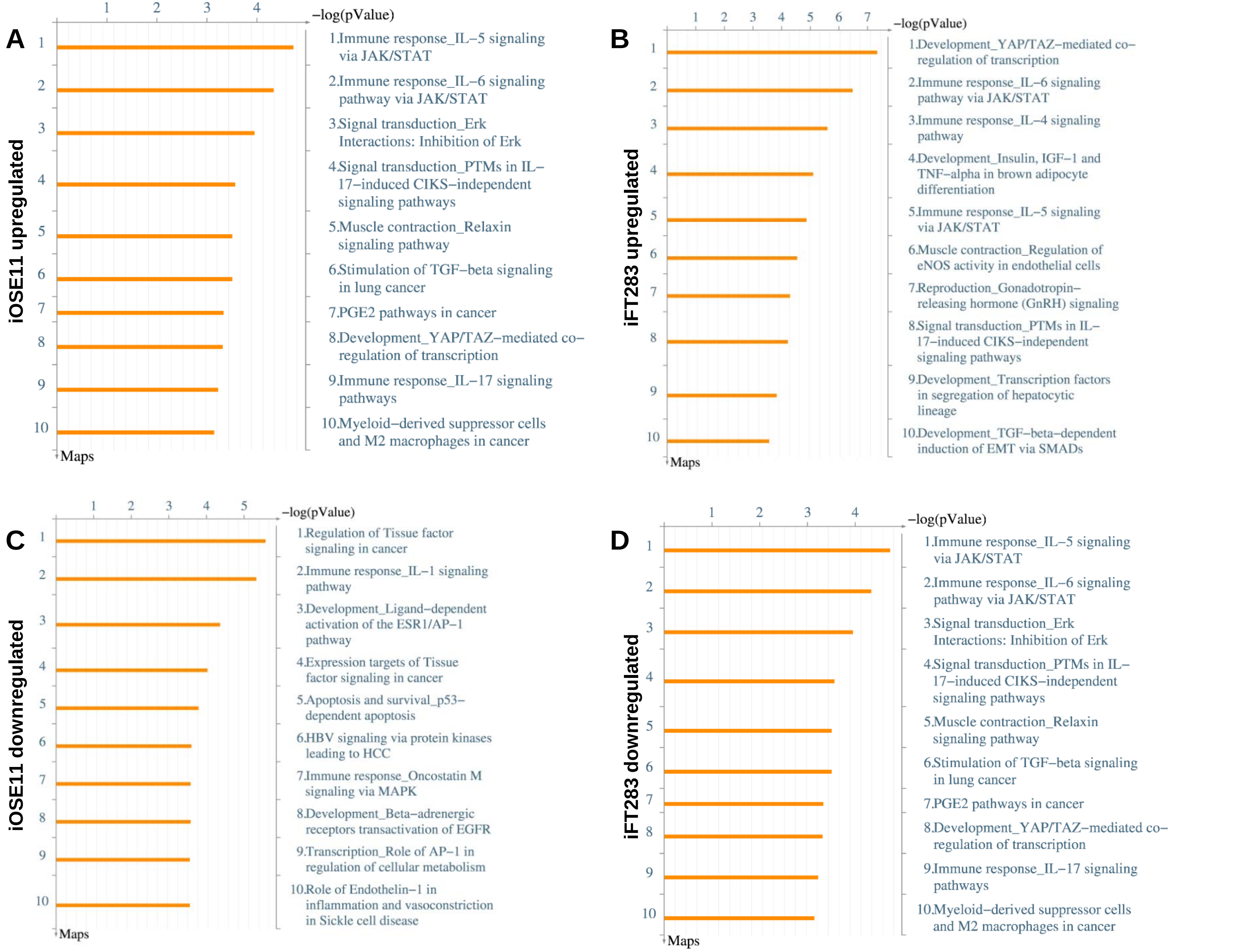

Supplementary Figure 4

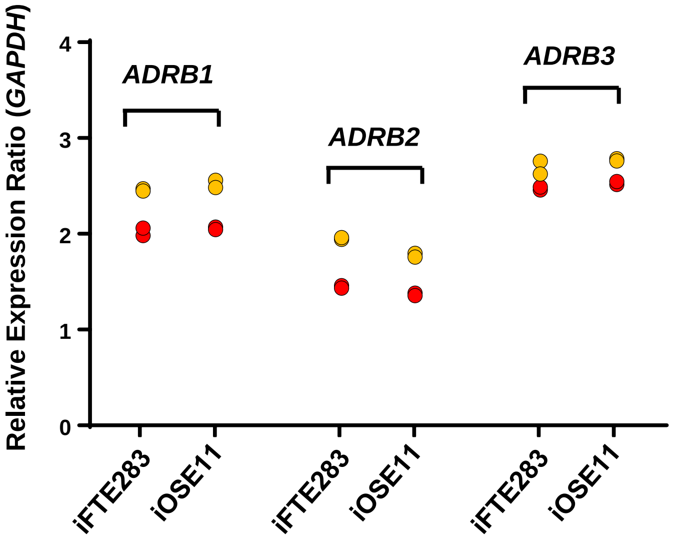

Supplementary Figure 5

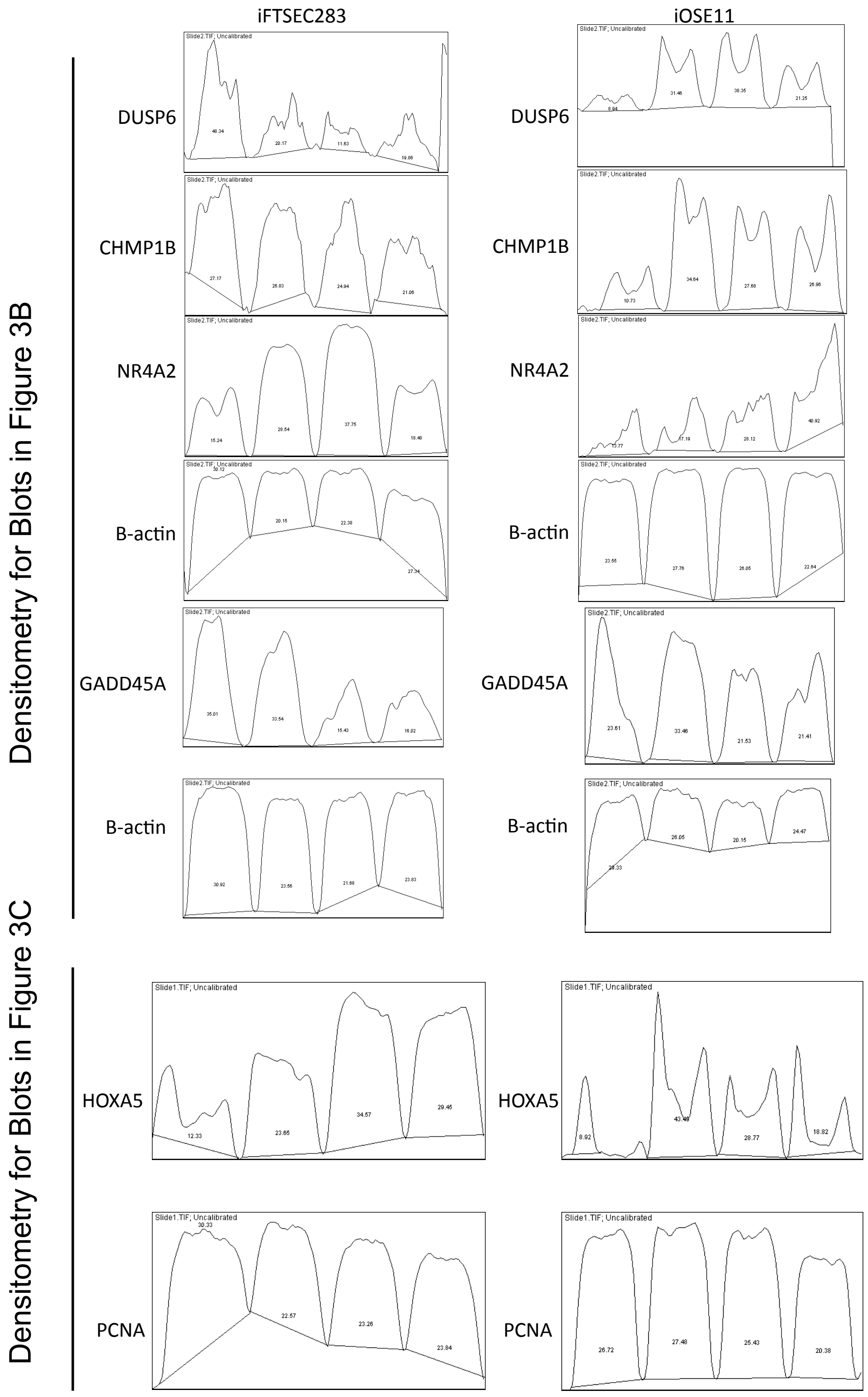

	FTSEC283					iOSE11				
10 uM NE Treatment	DUSP6	CHMP1B	NR4A2	GADD45A	HOX5A	DUSP6	CHMP1B	NR4A2	GADD45A	HOX5A
0	1	1	1	1	1	1	1	1	1	1
15'	0.41722	0.987706	1.872998	0.95815	1.918472	3.521038	3.229629	1.248149	1.417231	4.875126
1 h	0.240613	0.91821	2.477619	0.440867	2.804494	4.291629	2.580459	2.04131	0.911852	3.225199
4 h	0.41091	0.775021	1.212786	0.457636	2.389308	2.37791	2.513146	2.970597	0.906769	2.109068
